# Reshaping of the substrate-binding pocket to improve the catalytic activity of the non-specific endonuclease from *Pseudomonas fluorescens*

**DOI:** 10.3389/fbioe.2026.1834140

**Published:** 2026-05-29

**Authors:** Jing-Yi Zhang, Li-Jian Zhou, Qiang Zhou, Wen-Jie Si, Yue Wu, Ye-Wang Zhang

**Affiliations:** 1 School of Pharmacy, Jiangsu University, Zhenjiang, China; 2 The People’s Hospital of Danyang, Affiliated Danyang Hospital of Nantong University, Zhenjiang, China

**Keywords:** catalytic activity, molecular docking, non-specific endonuclease, protein engineering, site-directed mutagenesis

## Abstract

Non-specific endonuclease cleaves the phosphodiester bonds of DNA and RNA. In this study, the structure of the recombinant non-specific endonuclease from *Pseudomonas fluorescens* (*Pf*Nuc) was analyzed and engineered to improve the catalytic performance. A semi-rational engineering approach was used to optimize the structure of *Pf*Nuc, thereby enhancing its catalytic efficiency. The catalytic domain was analyzed to screen critical residues for mutation using multiple sequence alignments and molecular docking. Four residues were selected for site-directed mutagenesis, and the mutants were characterized to assess their catalytic characteristics. Through iterative mutagenesis, a combinatorial library of 15 mutants was constructed. Thirteen of the mutants exhibited varying degrees of improvement in catalytic characteristics. Among these mutants, D76T/S217G showed a 2.3-fold increase in DNA cleavage activity, while D76T/A136S/S142A/S217G achieved a 1.8-fold enhancement in RNA cleavage activity, compared to the wild-type. Molecular docking revealed that the activity enhancement is attributed to an expanded active pocket and hydrogen-bonding networks formed between mutant residues and substrates, thereby accelerating product dissociation from the active site.

## 1 Introduction

Nucleases belong to the metalloenzyme superfamily and are defined as a group of hydrolases capable of hydrolyzing the phosphodiester bonds of nucleic acids (Li and Rohrmann, 2000). Based on the location of the cleavage sites, they are classified as exonucleases that hydrolyze terminal bonds, or endonucleases that cleave internal bonds. Endonucleases are further categorized as sequence-specific or non-specific based on their sequence recognition specificity (Nilsen et al., 2010). Among them, non-specific endonucleases, which cleave DNA and RNA without sequence specificity (Yang, 2011), have emerged as indispensable tools for eliminating nucleic acid contaminants in biopharmaceutical manufacturing and molecular biology applications (Schmitz et al., 2019; Schwardmann et al., 2019; Asano et al., 2021; Khalifa et al., 2021). The pursuit of a desired non-specific nuclease that combines high catalytic activity, exceptional stability, and cost-effective production has been receiving a lot of attentions (Li and Rohrmann, 2000; Greenstone et al., 2012; Li et al., 2014).

The non-specific endonuclease from *Serratia marcescens* (*Sm*Nuc) represents the archetype and most widely used enzyme among non-specific endonucleases (Eaves and Jeffries, 1963; Ball et al., 1987; Benedik and Strych, 1998; Song and Zhang, 2008; Galieva and Filimonova, 2011). The crystal structure of *Sm*Nuc has been obtained by X-ray diffraction (Miller et al., 1994). It reveals a conserved metal-ββα domain that coordinates a Mg^2+^ ion to form the catalytic core (Pingoud et al., 2009; Romanova et al., 2017; Schmitz et al., 2020). This domain features a highly polarized surface, facilitating substrate recruitment, and contains four critical, highly conserved residues (Arg57, His89, Asn119, and Glu127) essential for the catalysis (Ball et al., 1992; Wang et al., 2006). The previous reports indicated that His89 acts as a general base (Kolmes et al., 1996; Khamidullina et al., 2017), while Arg57 and Asn119 stabilize the transition state (Miller et al., 1994; Friedhoff et al., 1996), with Asn119 also participating in metal ion coordination (Antosiewicz et al., 1997; Lunin et al., 1997). While non-specific nucleases have been investigated in a variety of strains, they continue to suffer from the disadvantages of high production costs for DNA and RNA (Fujimoto et al., 1974; Muro-Pastor et al., 1992; Varrot et al., 2000; Tang et al., 2008), poor storage stability, and low catalytic activity, which have restricted their widespread use in industrial applications.

To overcome these limitations, researchers have performed two complementary approaches: structural engineering of the existing enzymes and the exploration of novel natural enzymes. Alanine-scanning mutagenesis of *Sm*Nuc has rigorously validated the functional indispensability of its core catalytic residues (Gimadutdinow et al., 2018), providing a blueprint for rational design. Concurrently, the discovery of the non-specific endonuclease from *Pseudomonas fluorescens* (*Pf*Nuc) offered a promising alternative. In our previous work, *Pf*Nuc was successfully cloned and heterologously expressed in *Escherichia coli* (Han et al., 2024), which exhibited exceptional storage stability and strong substrate affinity although the enzyme showed high specific activity toward RNA and DNA.

In the present work, structural analysis and homologous sequence alignment were performed, and four residues proximal to the substrate-binding pocket were mutated to reshape the pocket for enhancing the catalytic activity of the non-specific endonuclease *Pf*Nuc. The constructed mutant library (15 mutants) encompassed all possible combinatorial mutations at four active-site positions. A comprehensive biochemical characterization assessed the catalytic activity, pH dependence, and thermal stability profiles of all mutants. Molecular docking simulations were used to elucidate the enzyme-substrate interactions and conformational changes.

## 2 Materials and methods

### 2.1 Materials and reagents

Yeast ribonucleic acid (RNA) and phosphomolybdic acid were from Solarbio (Beijing, China). Salmon sperm deoxyribonucleic acid (DNA) and kanamycin sulfate were purchased from Macklin (Shanghai, China). Agarose, agar powder, tryptone, and yeast dip were purchased from Sinopharm. The nickel-nitrilotriacetic acid (Ni–NTA) column, site-directed mutagenesis Kits, *E. coli* DH5α competent cells, *E. coli* BL21 (DE3) competent cells, SanPrep small mass DNA extraction Kit, and Isopropyl-D-β-thiogalactopyranoside (IPTG) were all obtained from Sangon Biotech (Shanghai, China). All other reagents were of analytical grade and commercially supplied.

### 2.2 Homology modeling

The amino acid sequence of non-specific endonucleases derived from *P. fluorescens* was analyzed using the BLAST (https://blast.ncbi.nlm.nih.gov/Blast.cgi). The theoretical structure of *Pf*Nuc was generated by homology modeling using the online tool SWISS-MODEL (https://swissmodel.expasy.org/) (Shlyapnikov et al., 2000; Waterhouse et al., 2018). Based on the high similarity (73.3%) between *Pf*Nuc and the non-specific endonuclease from *S. marcescens* (*Sm*Nuc, PDB code: 1G8T), the crystal structure of *Sm*Nuc was selected as the template for the modeling process. The models were evaluated using the PROCHECK on the SAVES online server (version 6.0: https://saves.mbi.ucla.edu/) (Colovos and Yeates, 1993).

### 2.3 Construction of the mutation library and site-directed mutagenesis

The amino acid sequence of non-specific endonucleases derived from *P. fluorescens* was analyzed using the BLAST (https://blast.ncbi.nlm.nih.gov/). The multiple sequence alignments of non-specific endonucleases obtained from a range of organisms were performed with ClustalW (https://www.genome.jp/tools-bin/clustalw), and the resulting alignments were visualized with Espript3.0 (https://espript.ibcp.fr/ESPript/). The mutation library was constructed by combining considerations of the substrate pocket and consensus design.

According to codon preferences in *E. coli*, the mutation primers of pET28a-*Pf*Nuc designed by PrimerX (http://www.bioinformatics.org/primerx/) are listed in Table 1. Site-directed mutagenesis kits were used to construct all single-point mutants. The full-length plasmids for each mutant were subsequently amplified by polymerase chain reaction (PCR) with the corresponding primers. After confirmation of the sequencing, the PCR products were transformed into *E. coli* BL21 (DE3) cells for overexpression.

**TABLE 1:** Design primers for mutation of *Pf*Nuc.

Primers	Sequence (5′to 3′direction)	T_m_ (^o^C)
D76T-F	CCG​GCG​AGC​GGT​AAAACCCGT​AAC​TGG​AAA​ACC	65.80
D76T-R	GGT​TTT​CCA​GTT​ACGGGTTTT​ACC​GCT​CGC​CGG	65.80
A136S-F	CAT​CAC​CCC​GCA​GAA​AAGCGAT​CTG​AAC​CAG​GGT​AG	67.70
A136S-R	CTA​CCC​TGG​TTC​AGATCGCTT​TTC​TGC​GGG​GTG​ATG	67.70
S142A-F	GAT​CTG​AAC​CAG​GGTGCATGG​GCG​CGT​CTG​GAA​G	68.10
S142A-R	CTT​CCA​GAC​GCG​CCC​ATGCACC​CTG​GTT​CAG​ATC	68.10
S217G-F	GAA​CAC​CCC​GAA​AGGTGCG​GAT​TTC​TGC​C	65.40
S217G-R	GGC​AGA​AAT​CCG​CACCTTT​CGG​GGT​GTT​C	65.40

### 2.4 Expression and purification of *Pf*Nuc and mutants

The recombinant *E. coli* containing the *Pf*Nuc gene or mutants were cultivated overnight at 37 °C, 220 rpm in 5 mL of Luria Bertani broth medium (50 μg⋅mL^-1^ kanamycin). The cells were transferred to 150 mL broth medium at 37 °C, 220 rpm until the OD_600_ reached 0.7–0.9. Isopropyl β-D-1-thiogalactopyranoside (IPTG) was added to a final concentration of 0.5 mM at 20 °C for induction of the protein expression. After 10 h of cultivation, the cells were harvested by centrifugation at 2300×g for 5 min at 4 °C and then washed with washing buffer (100 mM HEPES, pH 7.5 with 40 mM imidazole). After that, the cells were resuspended in lysis buffer (100 mM HEPES, pH 7.5) and ultrasonicated for 10 min. To harvest the soluble enzyme, the supernatant was collected after centrifugation (8,000 rpm, 10 min) to remove cell debris. The crude enzyme solution was loaded onto the equilibrated Ni-NTA resin column for binding for 3 h at 4 °C. The unbound proteins were rinsed with the washing buffer. The recombinant enzyme was collected with the elution buffer (100 mM HEPES, pH 7.5, containing 250 mM imidazole). The protein concentration of all the samples was measured by the Bradford method (Bradford, 1976). The purified *Pf*Nuc and the mutants were analyzed with 12% sodium dodecyl sulfate polyacrylamide gel electrophoresis (SDS-PAGE) to assess the purity.

### 2.5 Enzyme assay

One unit enzyme activity of non-specific endonuclease is defined as the amount of enzyme required to increase the absorbance at 260 nm in 30 min at pH 8.0 and 37 °C (Fujimoto et al., 1974; MacLellan and Forsberg, 2001). Salmon sperm DNA and yeast RNA were used as the substrates to determine the specific activities. The 1 mL reaction system contained 200 mM Tris-HCl buffer (pH 8.0), 5 mM MgCl_2_, 250 μg of *Pf*Nuc, and 10 mg of the substrate (DNA or RNA). After mixing thoroughly, the reaction was carried out at 37 °C for 15 min. And 100 μL of samples were withdrawn from the reaction system every 5 min, and added to 200 μL nucleic acid precipitation reagent (containing 0.25% perchloric acid solution and 2.5% phosphomolybdic acid). After incubation at 4 °C for 10 min, the mixture was centrifuged at 6000×g for 15 min. The absorbance of the supernatant at 260 nm was measured to calculate the enzyme activity according to the following formula:

$$Specific\;activity\;\left(U\cdot {mg}^{-1}\right)=\frac{{\Delta A}_{260}}{tm}$$

In the equation, ${\Delta A}_{260}$ is the value of absorbance change at 260 nm; *t* is the sampling time (min); *m* is the amount of residual enzyme in the reaction system (mg).

### 2.6 Enzymatic characterization

Effect of pH on the enzyme activity was measured in a range of pH from 4.0 to 9.0 with two buffer systems: 200 mM pH 4.0–5.8 acetate-sodium acetate buffer and 200 mM pH 7.1–9.0 Tris-HCl buffer. To investigate the effect of temperature on the enzyme activity, assays were conducted at the temperatures in a range of 25–70 °C under standard conditions. The thermostability of wild-type and the mutants was monitored by measuring the residual activity after incubation for different periods. To determine the t_1/2_ of *Pf*Nuc and its mutants, the catalytic activity of the wild type and its mutants was assessed after different incubation times at 37 °C (200 mM Tris-HCl, pH 8.0). To quantify activity loss for different times, the initial enzyme activity was set at 100%. Three independent replicates were performed, and standard deviations were calculated. The thermal denaturation temperatures T_m_ of *Pf*Nuc and its mutants were measured with the differential scanning calorimeter (DSC 25, TA, United States of America). Protein samples of WT and mutants were diluted to a final concentration of 1 mg/mL. Following equilibration at 20 °C, the temperature was increased from 25 °C to 90 °C at a rate of 5 °C/min. The change of heat flow was analyzed by TRISO 5.6, and the T_m_ was calculated accordingly.

Kinetic parameters of *Pf*Nuc and its mutants were determined by measuring the enzyme activity in 200 mM Tris-HCl buffer (pH 8.0, 5 mM Mg^2+^) at 37 °C with different substrate concentrations ranging from 1 to 60 mg⋅mL^-1^. The Michaelis constant (*K*_m_) and maximum velocity (*V*_max_) of the wild type and mutants were obtained by non-linear fitting of the Michaelis-Menten equation.

### 2.7 Molecular docking of DNA and RNA to the enzyme and mutants

The structures of the substrates, single-stranded RNA (ssRNA, 5′-GCUA-3′) and double-stranded DNA (dsDNA, 5′-ATAG-3’; 5′-CTAT-3′), were downloaded from PubChem (https://pubchem.ncbi.nlm.nih.gov/). Before molecular docking, the initial structural models of *Pf*Nuc and substrates were preprocessed to remove redundant water molecules and heteroatoms, repair incomplete side chains, and assign physiological protonation states. The AutoDock VINA (http://autodock.scripps.edu) was used to dock the substrate into the predicted models from Section 2.2 (Trott and Olson, 2009), with the gate position set to 0.5 Å. The coordinates of the box were centered at (x, y, z) = (4, 15, −6), and the box size was set as 50 × 50 × 50 Å. Energy minimization of the protein–nucleic acid complex was performed using GROMACS with the AMBER14SB force field and TIP3P water model, employing a two-step optimization strategy. The optimal complex conformation was selected comprehensively based on binding energy and conserved interaction characteristics of the active pocket. The first stage adopted the steepest descent algorithm with 5,000 steps, and a step size of 0.01, and the calculation stopped when the maximum force was lower than 1000 kJ/(mol·nm). Subsequently, the conjugate gradient method was applied for further optimization, with 5,000 steps, and a step size of 0.01, and the iteration was terminated at a threshold of 100 kJ/(mol·nm). A backbone constraint was applied during optimization to prevent unreasonable structural distortion and ensure the rationality and stability of the initial model and docking complex. The interactions were visualized with PyMOL (version 2.5).

Analysis of the substrate-binding cavity was performed on the modeled structures of *Pf*Nuc and its mutants to estimate its volume and surface area using the parKVFinder plugin in PyMOL (Guerra et al., 2020). The amino acid residues within 5 Å of the substrate in the catalytic pocket were used to define the region for parKVFinder calculation. Meanwhile, the following parameters were applied: Resolution = Low, Probe In = 1.4 Å, Probe Out = 10.0 Å, Removal Distance = 0 Å.

## 3 Results and discussion

### 3.1 Site-directed mutagenesis of *Pf*Nuc

**FIGURE 1:**
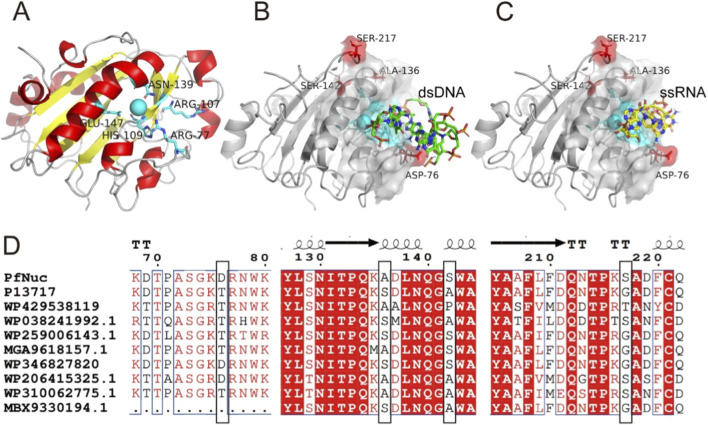
Selection of mutation residues to enhance catalytic activity. **(A)** Structure of *Pf*Nuc. The catalytic pocket with dsDNA **(B)** or ssRNA **(C)**, highlighting potential mutation residues in red and the catalytic center in blue. **(D)** Multiple sequence alignment of non-specific endonucleases.

The spatial structure of *Pf*Nuc obtained from the SWISS-MODEL is shown in Figure 1A. The catalytic center composes a typical Mg^2+^-dependent ββα domain (Wu et al., 2020). There are five highly conserved amino acid residues in the catalytic domain shown in blue, Arg77, Arg107, His109, Asn139 and Glu147, which are shown in blue and strictly conserved in the homologous enzyme *Sm*Nuc. The previous studies of *Sm*Nuc have demonstrated that these residues play critical roles in cleaving the phosphodiester bond (Friedhoff et al., 1996). This center features a pronounced positive electrostatic potential surface, which facilitates the binding and orientation of the negatively charged nucleic acid substrates. The catalytic domain of *Pf*Nuc, consistent with the structural feature of *Sm*Nuc, can accommodate both DNA and RNA. Molecular docking models visualizing these interactions are shown in Figures 1B,C. Structural analysis identified 42 residues located within 5 Å of the substrate-binding domain, which are colored gray in Figures 1B,C. Multiple sequence alignment revealed a high degree of conservation among these residues. Thus, the less conserved residues were selected in the present work as the targets for mutagenesis, as shown in Figure 1D. Four amino acid residues were chosen as mutation sites, including Asp76, Ala136, Ser142, and Ser217. Based on the multiple sequence alignment, highly conserved amino acid residues were selected for substitution. Finally, a focused mutation library with a total of 15 mutants was constructed, containing four single mutants (D76T, A136S, S142A, S217G), six double mutants (D76T/A136S, D76T/S142A, D76T/S217G, A136S/S142A, A136S/S217G, S142A/S217G), four triple mutants (D76T/A136S/S217G, D76T/A136S/S142A, D76T/S142A/S217G), and one quadruple mutant (D76T/A136S/S142A/S217G).

### 3.2 Heterologous expression and purification of *Pf*Nuc

**FIGURE 2:**
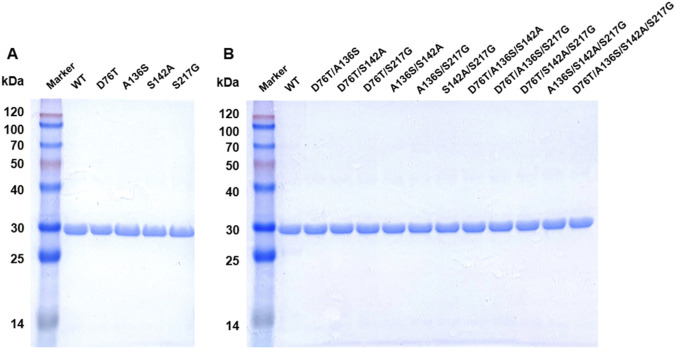
SDS-PAGE image of purified *Pf*Nuc mutants: **(A)** WT and single mutants. **(B)** WT and iterative mutants.

All 15 mutants were successfully expressed in *E. coli*. Following expression, the *Pf*Nuc and mutants were purified from cultured cells with Ni-NTA affinity chromatography and analyzed with SDS-PAGE. Both WT and mutants show a clear band at the expected position of 29 kDa in Figure 2.

### 3.3 Determination of the catalytic activity

**FIGURE 3:**
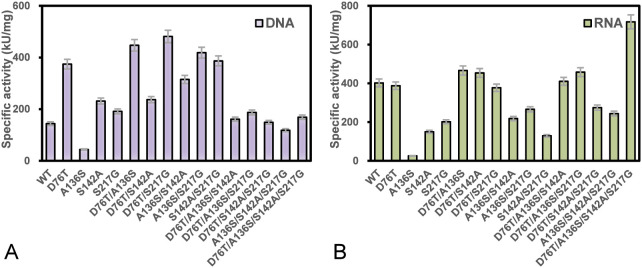
The catalytic activity of the wild-type *Pf*Nuc and its mutants for DNA **(A)** and RNA **(B)**.

The specific activities of wild-type *Pf*Nuc and its mutants were characterized using salmon sperm DNA and yeast RNA as the substrates. As shown in Figure 3, the wild-type *Pf*Nuc exhibited specific activities of 1.44 × 10^5^ U⋅mg^-1^ and 4.02 × 10^5^ U⋅mg^-1^ for DNA and RNA, respectively. Among the single-point mutants, D76T, S142A, and S217G exhibited higher DNA activity, which were 2.6-, 1.6-, and 1.3-fold that of the wild-type, respectively, while A136S severely impaired activity toward both substrates (DNA: 30.6%; RNA: 6.4%). These four mutants for RNA activity diverged: D76T retained most of the wild-type RNA activity (96.4%), whereas S142A and S217G decreased to 37.3% and 50.1%. With the significantly enhanced DNA activity while maintaining RNA activity, D76T was identified for further engineering.

The 11 iterative mutants were constructed based on four single-point mutants. All double mutants showed further improved DNA activity. The mutant D76T/S217G was the most notable one, exhibiting 3.3-fold higher DNA activity than the wild-type while retaining 93.8% of the RNA activity. The mutant D76T/A136S displayed apparent enhanced catalytic activity, with 3.1-fold higher DNA activity and a slight increase in RNA activity (115.9%). These two mutants displayed improved DNA catalytic efficiency without compromising RNA activity. In contrast, other mutants including A136S/S142A, A136S/S217G, S142A/S217G showed enhanced DNA activity but severely reduced RNA activity. Compared to the double mutants with significant DNA activity advantages, the triple and quadruple mutants showed a lower enhancement in DNA activity, ranging from 82% to 130% of the wild-type level. Among them, the mutant D76T/A136S/S217G retained significant activity for both DNA and RNA, at 130% and 114% of the wild type. Remarkably, the mutant D76T/A136S/S142A/S217G exhibited a distinct shift in substrate preference: its RNA activity reached 178% of the wild-type, the highest among all tested mutants. In comparison, its activity to DNA (117%) was comparatively lower than that of the double mutants.

The kinetic parameters of the mutants with enhanced activity, namely, D76T, D76T/A136S, D76T/S217G, D76T/A136S/S217G, and D76T/A136S/S142A/S217G, were determined and summarized in Table 2. The catalytic efficiency of these mutants toward DNA was significantly increased compared with the wild-type, which was primarily reflected in elevated *V*_max_ ranging from 1.2 to 1.6-fold of WT. Notably, the D76T/A136S mutant exhibited enhanced substrate affinity for DNA, with a *K*_*m*_ of 12.9 mg·mL^-1^ compared to 14.2 mg·mL^-1^ for the wild type. Regarding RNA catalysis, the kinetic characteristics exhibited enhanced activity: mutants D76T/A136S/S217G and D76T/A136S/S142A/S217G maintained *V*_max_ comparable to (94.7%) or higher than (123.6%) than that of WT, consistent with the increased RNA-cleaving activity. In contrast, mutants D76T, and D76T/S217G showed a lower *V*_max_ for RNA, which is consistent with their decreased catalytic activity.

**TABLE 2:** Kinetic parameters of wild-type *Pf*Nuc and its mutants.

Kinetic parameters	*V*_max_ (U·mg^-1^)		*K*_*m*_ (mg·mL^-1^)		*K*_*cat*_/*K*_*m*_ (mL·(mg·s)^−1^)	
Substrate	RNA	DNA	RNA	DNA	RNA	DNA
WT	1.1 × 10^6^	6.4 × 10^5^	14.2	14.2	1.43 × 10^4^	8.19 × 10^3^
D76T	9.5 × 10^5^	8.5 × 10^5^	14.4	16.2	1.20 × 10^4^	9.55 × 10^3^
D76T/A136S	1.6 × 10^6^	7.6 × 10^5^	15.1	12.9	1.90 × 10^4^	10.80 × 10^3^
D76T/S217G	9.2 × 10^5^	1.0 × 10^6^	9.9	18.1	1.69 × 10^4^	10.40 × 10^3^
D76T/A136S/S217G	1.1 × 10^6^	7.7 × 10^5^	10.7	14.2	1.80 × 10^4^	9.94 × 10^3^
D76T/A136S/S142A/S217G	1.4 × 10^6^	8.0 × 10^5^	15.4	15.7	1.63 × 10^4^	9.36 × 10^3^

### 3.4 Effect of pH on catalytic activity

**FIGURE 4:**
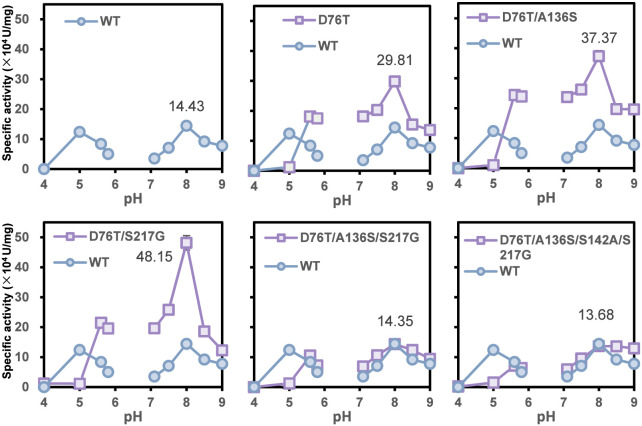
Effect of pH on the DNA catalytic activity of *Pf*Nuc mutants.

**FIGURE 5:**
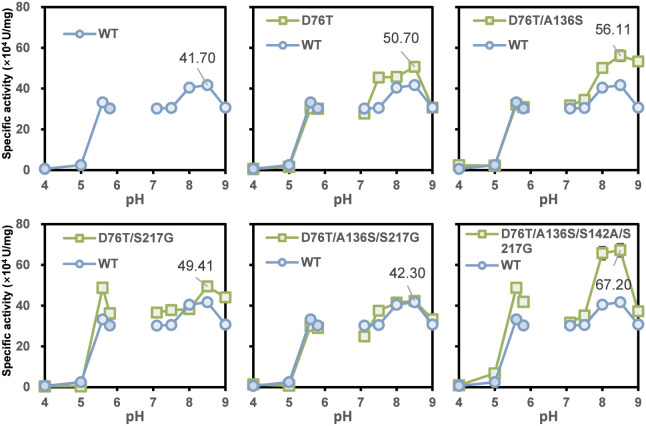
Effect of pH on the RNA catalytic activity of *Pf*Nuc mutants.

To assess whether mutations at key residues affect the active site architecture or pH dependence of catalysis, the activities of wild-type and its mutants were systematically determined across a range of pH conditions. As shown in Figures 4, 5, all mutants fully retained the pH-dependent characteristics of the wild-type enzyme. Specifically, their optimal reaction pH remained identical to that of the wild type for both DNA (pH 8.0) and RNA (pH 8.5) substrates. The presence of a secondary activity peak at pH 5.6 across all variants defined a stable biphasic pH-activity profile. These findings indicate that the mutations did not substantially alter the protonation states of the active-site residues or the proton requirement of the catalytic center (Ramachandran et al., 2016).

### 3.5 Thermostability of wild-type *Pf*Nuc and its mutants

**FIGURE 6:**
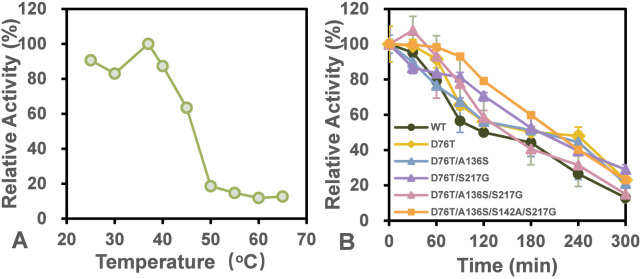
**(A)** Effect of temperature on the relative activity of *Pf*Nuc for DNA. **(B)** The thermostability of *Pf*Nuc wild type and mutants for DNA at 37 °C.

To assess the thermal stability of *Pf*Nuc, the DNA-based reaction system was selected for analysis. Since *Pf*Nuc shows identical *K*_m_ for both DNA and RNA, either substrate could be effectively used to monitor the heat-induced loss of structural integrity. DNA was chosen for practical reasons: it shows greater chemical stability than RNA during extended high-temperature incubation and offers better reproducibility under the current assay conditions. To investigate the effect of temperature on the enzyme activity, Figure 6 shows the activity profile of the wild-type enzyme across a temperature range of 25–70 °C. The highest activities for DNA were observed at 37 °C. The thermostability of wild-type *Pf*Nuc and its mutants was assessed by determining the t_1/2_ of their catalytic activity for DNA at 37 °C. All mutants demonstrated a 24%–75% increase in half-life over the wild type which had t_1/2_ of 120 min. Among them, the double mutant D76T/S217G showed a 60% increase, corresponding to a t_1/2_ of 192 min. The most significant enhancement was observed with the quadruple mutant D76T/A136S/S142A/S217G, which achieved a 75% increase and t_1/2_ of 210 min. Compared to the wild type, all tested mutants demonstrated higher thermal stability, as reflected by an increased T_m_. The observed Tm values were consistently elevated, showing a rise of approximately 2.80–11.26 °C over that of the WT (Table 3).

**TABLE 3:** The catalytic activity and thermostability of *Pf*Nuc wild type and mutants.

Enzymes	Specific activity-DNA (U·mg^-1^)	Specific activity-RNA (U·mg^-1^)	t_1/2_ (min)	T_m_ (^o^C)
WT	144000	402210	120	61.76
D76T	374400	387734	180	64.56
D76T/A136S	447200	466389	192	64.69
D76T/S217G	481500	377222	192	69.91
D76T/A136S/S217G	187341	457842	148	68.39
D76T/A136S/S142A/S217G	168912	717600	210	73.02

### 3.6 Molecular docking

**FIGURE 7:**
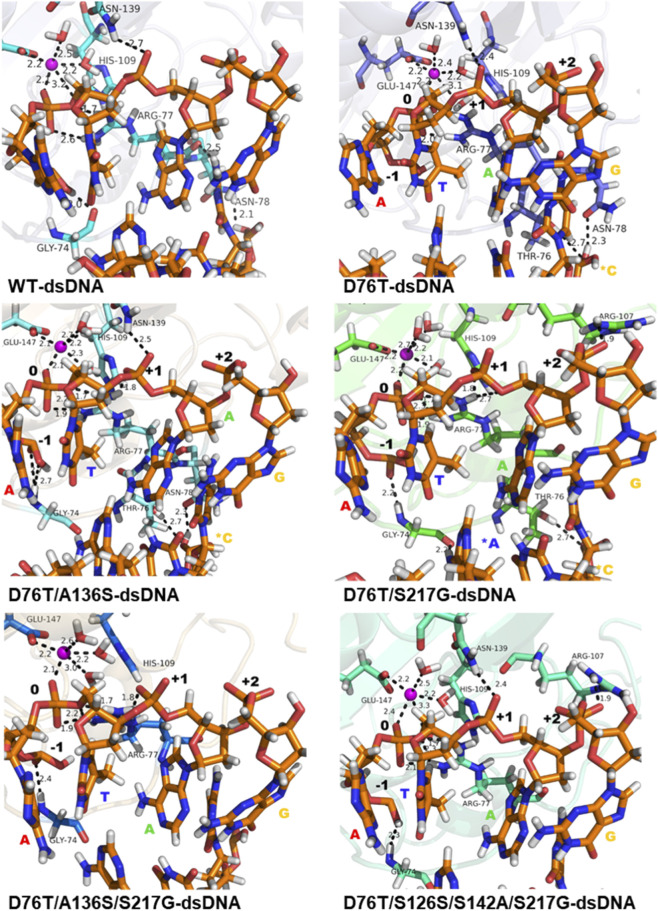
Molecular docking of dsDNA and *Pf*Nuc WT and the mutants. In the complex structure, dsDNA is shown with carbon atoms in orange, oxygen atoms in red, nitrogen atoms in blue, and hydrogen atoms in gray; the key amino acid residues are displayed with carbon atoms in distinct colors. All intermolecular hydrogen bonds between *Pf*Nuc and substrates were defined as hydrogen–acceptor distances. The Mg^2+^ mediates coordination with Glu, weak binding to water, and electrostatic interactions with nucleic acid phosphate oxygen.

**FIGURE 8:**
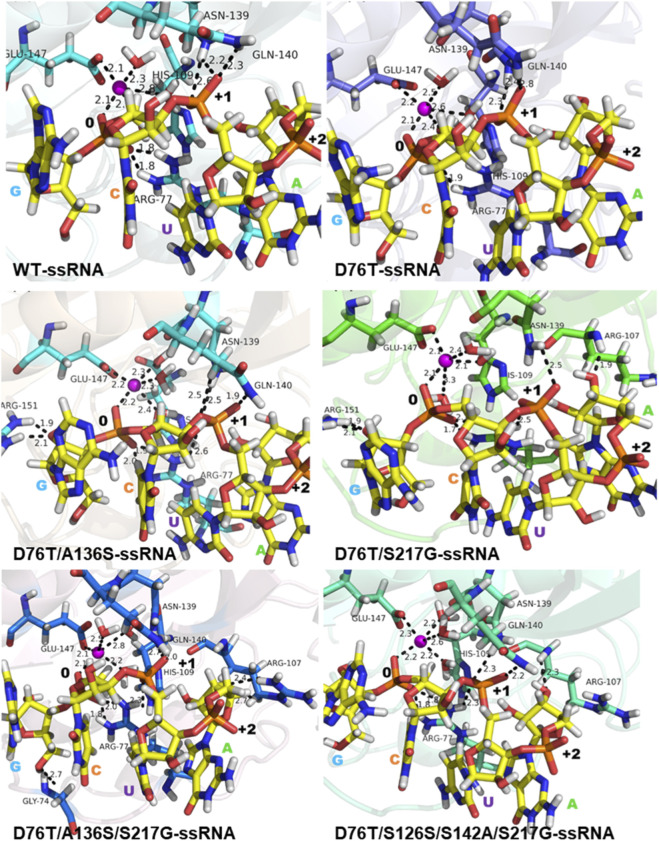
Molecular docking of ssRNA and *Pf*Nuc WT and the mutants. In the complex structure, ssRNA is shown with carbon atoms in yellow, oxygen atoms in red, nitrogen atoms in blue, and hydrogen atoms in gray; the key amino acid residues are displayed with carbon atoms in distinct colors. Hydrogen bond statistics and Mg^2+^ interaction patterns are consistent with Figure 7.

Molecular docking of *Pf*Nuc with dsDNA and ssRNA has been performed to explore the structural basis for the retained activity of the mutants (Figures 7, 8). Most intermolecular hydrogen bonds between *Pf*Nuc and substrates are dominated by nucleic acid backbone atoms, which is consistent with the non-specific cleavage property of *Pf*Nuc. The D76T/S217G mutant exhibited markedly enhanced activity toward DNA. The catalytic activity of the D76T/S217G was 334% of the wild type for DNA and 94% of the wild type for RNA. To investigate the structural basis of this shift, comparative molecular docking with dsDNA was performed. The results indicated that the mutant complex formed nine hydrogen bonds with an average length of 2.51 Å, compared to six bonds (2.26 Å) in the wild type, suggesting enhanced substrate affinity (Baker and Hubbard, 1984). This increased stabilization is primarily attributed to several newly formed or strengthened interactions. Gly74 formed two hydrogen bonds with the −1 phosphate group, potentially facilitating product release. The substitution of Asp76 with threonine enabled a new 2.70 Å hydrogen bond via its hydroxyl group, and Arg77 formed 5 hydrogen bonds, more than in the wild-type complex. Collectively, these structural rearrangements stabilize the enzyme-dsDNA complex and likely the phosphorane intermediate, thereby accounting for the observed enhancement in DNA cleavage activity (Friedhoff et al., 1996).

D76T/A136S/S142A/S217G displayed improved RNA activity, which was 1.8-fold that of the wild type. The wild-type enzyme formed 5 hydrogen bonds (average length: 2.14 Å) with the ssRNA substrate; the mutant also formed 5 hydrogen bonds with the ssRNA, but with a slightly shorter average bond length (2.12 Å), indicating a lower energy of the complex between the mutant and ssRNA. This enhanced stabilization might primarily be from the strengthened interactions at key residues. Arg77 forms 2.30 Å interactions with the phosphate group at position +1, in addition to 2 hydrogen bonds (with bond lengths of 1.80 and 1.80 Å) with the phosphate group at position 0. Similar to the mutant D76T/A136S/S217G-ssRNA binding conformation, Arg107 forms an interaction with the phosphate backbone, facilitating the entry of substrate molecules into the active pocket. Collectively, these refined interactions at Arg77 and Arg107 likely contribute to the observed increase in catalytic efficiency toward ssRNA.

**FIGURE 9:**
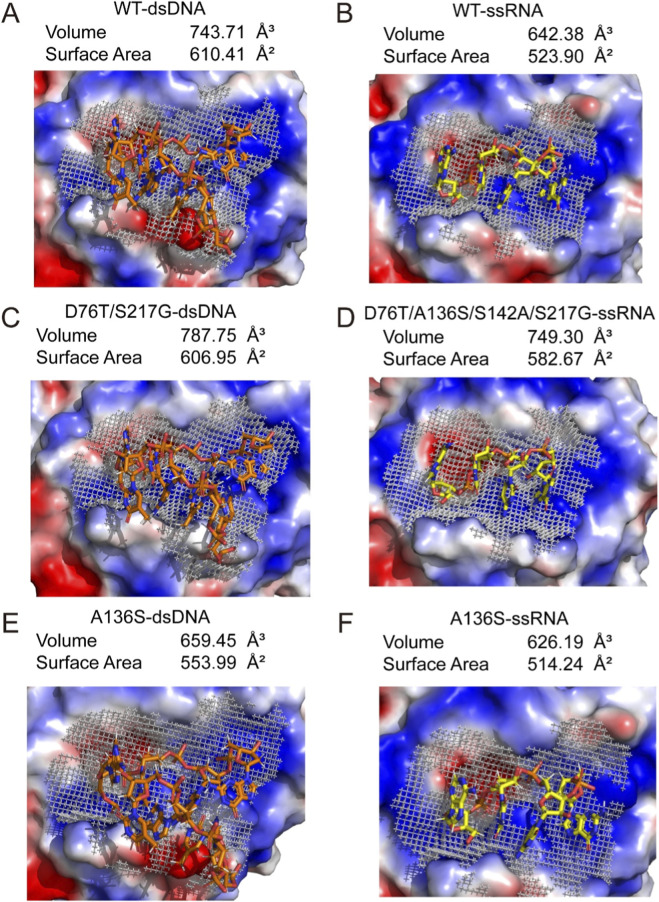
Calculation of the substrate-binding cavity volume and surface area of WT-dsDNA **(A)**, WT-ssRNA **(B)**, D76T/S217G-dsDNA **(C)**, D76T/A136S/S142A/S217G-ssRNA **(D)**, A136S-dsDNA **(E)**, A136S-ssRNA **(F)**.

The substrate-binding cavities of the wild-type and the two highly active mutants, D76T/S217G and D76T/A136S/S142A/S217G, were analyzed to elucidate the structural basis for their enhanced catalytic activity in Figure 9. Calculations using parKVFinder revealed a significant expansion in the volume of the catalytic cavity in both mutants (Saha et al., 2020). Specifically, the cavity volume of D76T/S217G-dsDNA increased by 5.92% (44.06 Å^3^), compared to WT-dsDNA. D76T/A136S/S142A/S217G-ssRNA exhibited an even more pronounced cavity expansion, with a volume increase of 16.64% (106.92 Å^3^). In contrast, the single-point mutant A136S-dsDNA displayed a notable reduction in substrate-binding cavity volume, with an absolute decrease of 84.26 Å^3^ relative to the wild-type structure. Meanwhile, the A136S-ssRNA complex also presented a decreased cavity volume, with a reduction of 16.19 Å^3^. This obvious contraction of the binding pocket compressed the internal spatial environment, restricted substrate insertion and proper orientation within the catalytic region, and disturbed the rational arrangement of key catalytic residues. These adverse structural alterations weakened substrate accessibility and binding capacity, thereby explaining the reduced catalytic activity of the A136S mutant. These structural changes provide direct structural evidence that the enlarged cavity promotes substrate entry and improved access to the catalytic center, including key residues His109, Glu147, and Mg^2+^.

Analysis of the cavity surface area further indicated remodeling of the binding interface. For the ssRNA substrate, the surface area of the D76T/A136S/S142A/S217G-ssRNA increased by 11.21% (58.77 Å^2^), relative to WT. This indicates that the cavity expansion likely involves a reshaping of the interior surface to fit the substrate, rather than just a uniform size increase. Notably, both high-activity mutants share the D76T substitution, which is located at a critical position for dsDNA binding. The replacement of Asp with Thr may play a key role in modulating local conformation to enable cavity enlargement. Notably, both high-activity mutants share the D76T substitution, which is located at a critical position for dsDNA binding. The replacement of Asp with Thr may play a key role in modulating local conformation to enable cavity enlargement.

## 4 Conclusion

The catalytic activity of a-non-specific endonuclease derived from *P. fluorescens* was improved through a semi-rational design strategy. Notably, the engineered mutant D76T/S217G exhibited a 334.4% increase in catalytic activity to DNA compared to the wild-type enzyme, and the mutant D76T/A136S/S142A/S217G exhibited a 178.4% increase to RNA. Computational and structural studies suggest that the enhanced activity arises from optimized hydrogen-bond networks and stabilized substrate-transition state interactions. Analysis of the binding pocket indicated that the enhanced catalytic activity of the mutant is also associated with a significantly enlarged pocket volume during product release.

## Data Availability

The datasets presented in this study can be found in online repositories. The names of the repository/repositories and accession number(s) can be found below: https://www.uniprot.org/, 1g8t.
